# Successful percutaneous coronary intervention in the setting of an aberrant left coronary artery arising from the right coronary cusp in a patient with acute coronary syndrome: a case report

**DOI:** 10.1186/s12872-017-0621-3

**Published:** 2017-07-11

**Authors:** Jung-Hee Lee, Jong-Seon Park

**Affiliations:** Division of Cardiology, Yeungnam University Medical Center, Yeungnam University College of Medicine, 3170, Hyeonchung-ro, Nam-gu, Daegu, Korea

**Keywords:** Coronary anomaly, Acute coronary syndrome, Percutaneous coronary intervention

## Abstract

**Background:**

An aberrant origin of the left coronary artery (LCA) from the right coronary cusp (RCC) is an extremely rare congenital anomaly. We here report on successful percutaneous coronary intervention (PCI) in a patient presenting with acute coronary syndrome and an aberrant origin of the LCA from the RCC.

**Case presentation:**

A 50-year-old man presented at our emergency department with recurrent resting chest pain. Following unsuccessful attempts at visualizing the left coronary artery using Judkins left and Amplatz catheters, an aortogram using a pigtail catheter suggested anomalous left coronary artery origin and showed a significant occlusive lesion at proximal left anterior descending artery. A Judkins right 4 guiding catheter was placed around the left coronary ostium and exchanged for a Judkins left 3.5 guiding catheter after introducing a .014" coronary long wire into the left circumflex artery. With excellent angiographic visualization and guide support, a drug-eluting stent was then successfully implanted. Cardiac computed tomography (CT) demonstrated left coronary artery origin from right coronary cusp.

**Conclusion:**

This report presents a case of LCA originating from the RCC accompanied with acute coronary syndrome that was treated with successful PCI.

## Background

Coronary artery anomalies are rare findings in about 1% to 2% of adults [1], with aberrant origin of the left coronary artery (LCA) from the right coronary cusp (RCC) being extremely rare (0.15% incidence) [2]. Furthermore, coronary artery anomalies presenting with acute coronary syndrome are also uncommon, and often are challenging to manage [3]. We report on successful percutaneous coronary intervention (PCI) in a patient with acute coronary syndrome and LCA originating from the RCC.

## Case presentation

A 50-year-old man with a medical history of hypertension, diabetes mellitus, and dyslipidemia presented to our emergency department complaining of recurrent resting chest pain (Canadian Cardiovascular Society Class III). A physical examination revealed that the patient’s blood pressure was 80/40 mmHg, his pulse rate was 61 beats per minute, and that he displayed an absence of any abnormal cardiac or respiratory sounds. An electrocardiography showed normal sinus rhythm without significant ST-T wave abnormality, and chest X-ray findings were unremarkable. His serum troponin I peaked at 0.09 mcg/L (ULN ≤0.05 mcg/L) within 24 h of presentation. Under the clinical diagnosis of unstable angina, he was treated in accordance with acute coronary syndrome guidelines [4, 5].

**Fig. 1:**
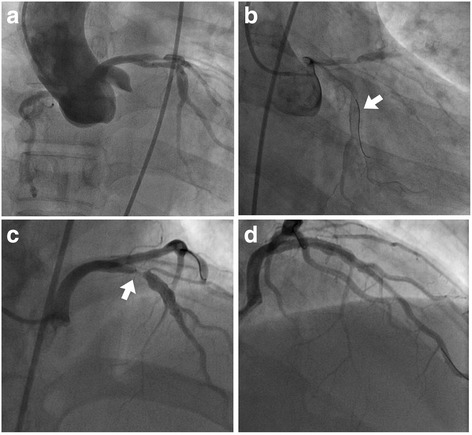
**a** Non-selective coronary angiography using a pig-tail catheter demonstrated anomalous origin of left coronary arteries and a significant occlusive lesion at the proximal left anterior descending artery. **b** With a Judkins right guiding catheter, a .014″ coronary long wire was introduced to the left circumflex artery (*arrow*). **c** For superior back-up support, the Judkins left guiding catheter was exchanged revealing significant stenosis at the left anterior descending artery (*arrow*). **d** Following percutaneous coronary intervention, there was no residual stenosis or dissections observed

**Fig. 2:**
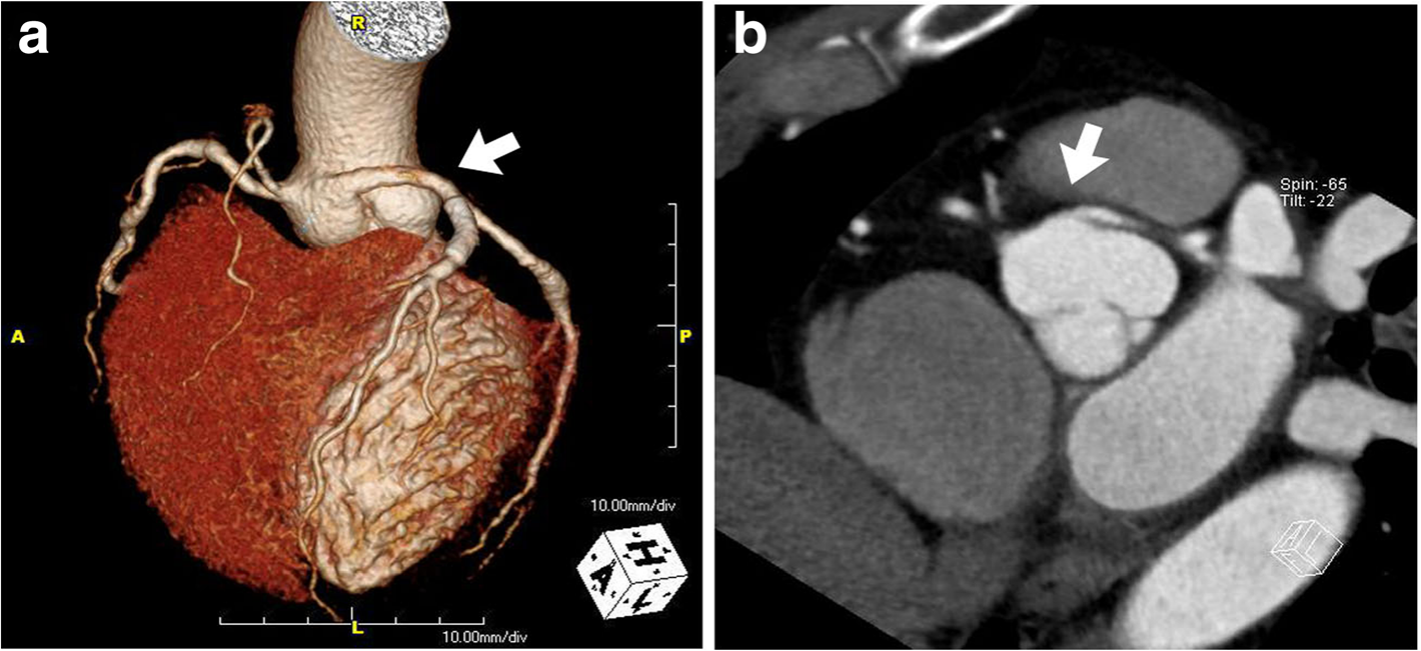
**a** Computed tomography coronary angiography revealed anomalous origin of the left coronary arteries from the right coronary cusp (*arrow*) in three-dimensional reconstructed image. **b** Computed tomography coronary angiography showed the anomalous origin of left coronary artery and traveling behind the pulmonary artery (*arrow*)

While using the femoral approach with a 6-Fr sheath, no luminal stenosis was apparent at the right coronary artery (RCA) in RCA angiography. Left main coronary artery could not be engaged with conventional diagnostic catheters, such as Judkins Left 4 and Amplatz 1.0, and nonselective angiography using pig-tail catheter raised suspicion that the left coronary artery (LCA) was originating from the right coronary cusp (RCC) with an up-to-90% occlusive lesion present at the proximal left anterior descending artery (LAD) (Fig. 1a). After a Judkins right 4 guiding catheter was placed around the left coronary ostium, a .014″ coronary long wire (RG3, Asahi-Intecc, Nagoya, Japan) was successfully introduced into the left circumflex artery (LCx). Because the Judkins right guiding catheter was too short to reach the left coronary ostium, it was exchanged with a Judkins left 3.5 guiding catheter (Fig. 1b) instead, which was deeply intubated into the left main coronary artery with ballooning support on the LCx wire. Following guiding catheter stabilization, angiography clearly revealed a tubular eccentric proximal LAD with 90% stenosis (Fig. 1c). After passing an .014″ coronary guide wire (Runthrough®, TERUMO Inc., Japan) through the lesion, balloon angioplasty was performed with a 3.0 × 15-mm balloon. A 4.0 × 18-mm drug-eluting stent (XIENCE Alpine®, Abbott Vascular, Santa Clara, CA, USA) was then implanted with adjuvant ballooning performed with a 4.5 × 10-mm balloon. After successful proximal LAD revascularization, final angiography showed no residual stenosis or complications (Fig. 1d). The patient tolerated the procedure well, and appeared in good condition postoperatively. Two days later, computed tomography coronary angiography to establish the LCA system course revealed an anomalous origin of LAD and LCx from the right sinus of Valsalva (Fig. 2).

## Discussion and Conclusions

The present report illustrates the use of an effective PCI approach in the setting of an aberrant LCA from the RCC, which is very rare, with a reported incidence of only 0.02% to 0.15% [1, 2, 6–8]. In the few cases reported from Korea, this clinical condition has been treated either with conservative care or open heart surgery [9, 10], and one with PCI in which all three vessels arose from the right coronary ostium and an Amplatz right guiding catheter was engaged into the ostium of the LCx [11, 12]. Shah et al. reported a case of PCI in a patient with an anomalous left main originating from the right sinus of Valsalva that was successfully treated with a Judkins Left 3.5 guide support [13]. Unlike previous cases, our patient showed the LCA from the RCC separately from the right coronary ostium being successfully treated with PCI.

Approximately 0.14% of anomalous coronary arteries originating from the opposite sinus of Valsalva showed significant vessel compression which could be associated with major adverse cardiac events [14]. Because of reported possible associations between coronary anomalies and serious adverse clinical events [15–18], the prompt diagnosis and management of an anomalous coronary artery origin is important during acute coronary syndrome, as underscored by the present case.

According to ACC/AHA guidelines, possible anomalous coronary artery origin from the opposite sinus of Valsalva should be identified for evaluation of aborted sudden cardiac death or life-threatening arrhythmia [19]. However, coronary artery anomalous origins are often found incidentally at the time of invasive coronary angiography. While conventional angiography has projectional vascular overlap limitations, ECG-gated multidetector CT coronary angiography allows for the accurate imaging of an anomalous coronary artery origin, course, and relationship with cardiac structures and major arteries [20–22]. As illustrated here, CT coronary angiography should be performed even after successful revascularization to detect malignant anomalous coronary origin from the opposite sinus of Valsalva.

In conclusion, we present a patient with anomalous LCA originating from the RCC accompanied with acute coronary syndrome, who underwent successful percutaneous coronary intervention.

## Abbreviations

CT: Computed tomography
LAD: Left anterior descending artery
LCA: Left coronary artery
LCx: Left circumflex artery
PCI: Percutaneous coronary intervention
RCA: Right coronary artery
RCC: Right coronary cusp
